# The Doctor, the Child and the Community

**Published:** 1905-03

**Authors:** Marion McH. Hull

**Affiliations:** Atlanta, Ga.; Visiting Physician to Presbyterian Hospital; Pediatrist to the Sheltering Arms


### THE DOCTOR, THE CHILD AND THE COMMUNITY.*

BY MARION McH. HULL, M.Sc., M.D.

Visiting Physician to Presbyterian Hospital; Pediatrist to the Sheltering Arms.

In every community there are many classes of society. While all classes are necessary, and therefore important, they vary in the degree of their importance to the community. That of greatest importance is not the preacher nor the teacher, the lawyer nor the laborer, the merchant nor the musician, nor the doctor, although doubtless each considers his respective class indispensable. The most important is the infant. There are communities that have lived and progressed without the preacher, the teacher, the lawyer, or the doctor, but never was there one that grew without the infant.

Upon the infant depends, not only the continued existence and growth of the community, but also its possibilities in the realm of nations. Wrapped up in that epitome of weakness is inestimable power; though ignorant itself, it has the possibilities of infinite knowledge; without influence, yet it shall wield an influence that eternity alone can measure. Happy is that community whose every home is brightened with the merry laughter of its children, for great are their possibilities and importance.

*Read before the Atlanta Society of Medicine, February 2, 1905.

These statements must be qualified, however, The child must be a vigorous one if the community is to be blessed by it. Without a strong body the tendency is to a defective mentality, and this does not offer the best hope for a strong morality. In fact, there is a very close relation between the physical condition of the child and the mental and moral tone of the man. In general terms, the strong, vigorous child will develop a strong mind and a positive morality. Not that every healthy boy will make a mental giant or a remarkably moral man; nor that every delicate child will grow up an imbecile and immoral; but that a strong body offers less resistance to the forces that make for the building up of vigorous mentality and morality than does a weak body, and that the weak body offers less resistance to the forces of evil than a strong one does. I doubt not but that the first statement will be accepted by all; but I anticipate that some exception might be taken to the development of the resultant morality or immorality respectively; therefore, let me explain more carefully. While fully recognizing the innate depravity of every child, and also the power of God to work in any of His creatures as he will, whatever their state, yet it is true that the probability of a man's mind being in a receptive mood and of his character being moulded into one of great moral strength depends largely upon his physical and mental health, and vice versa. For example (and we shall illustrate the latter), an accident of birth produces cerebral hemorrhages; the infant is subject to convulsions; this lack of power to inhibit reflexes develops into some form of epilepsy in the child; the man, with increase of power to inhibit reflexes, has outgrown the tendency to convulsions, but exhibits other defects of his nervous system, such as kleptomania, erotomania, dipsomania, etc. The lack of the moral tone of the man is the result of the poor physique of the child. This is an extreme case, but lesser ones are those of degree only.

Now, since the child is of such importance to the community, and since the physical condition of the child has so much to do with the mentality and morality of the man, let us consider some of the factors that enter into the make-up of the best type of physique. These are antenatal and postnatal.

The old saying that it takes fifty years to make a gentleman is but an acknowledgment of the influences that operate upon the life of the unborn child. We all recognize the effect of heredity, but I trow there is a deal more blamed upon this factor than should properly be saddled upon it. The hereditary influences that might, and do often, detract from a good physique are syphillis (directly transferred), gonorrhœa (indirectly by weakening the vitality of the spermatazoon or of the endometrium), tuberculosis and alcoholism. The latter is responsible for most of the cases of hereditary deaf-mutism and for a large percentage of the cases of hereditary epilepsy. This suggests the advisability of marital laws that would prevent the continuance of these conditions, but this is too large a question to discuss here, if this were the time.

There are also, however, certain maternal influences that bear upon the physical well-being of the offspring, reference being made particularly to those that should be brought to bear upon the mother before and during her pregnancy that would fit her for maternity. For example, there is an increasingly large number of women who are unable to nourish their children; statistics could be exhibited to prove that a very much larger percentage of breast-fed infants survive and thrive than of artificially fed infants, and that their growth and development are proportionately greater and better. The probability of an infant's living and developing into a strong and vigorous child is directly proportionate to the ability of the mother to properly nourish it. One antenatal factor, therefore, is the proper dress, exercise, and care of the woman who is called to the highest profession under heaven so that she may be able properly to perform the duties of her calling and help her child to obtain a vigorous body.

Another antenatal influence that must be considered is one of the increasing evils of today. To their shame be it said that there is an increasing number of women to-day who are unwilling for one cause or another to undertake the responsibilities of motherhood. The opinion of the authorities is that the means used to prevent conception undermine the health of a great many women, their reproductive organs become more or less diseased, and if conception should occur and the pregnancy be uninterrupted, the probabilities are that the offspring will either be actually unhealthy, or at least not as vigorous as it might have been if the mother's organs had been normal. A large proportion of the deformities, such as harelip, cleft palate, etc., are said to be due to disease of the mother's reproductive organs.

Difficulties of delivery are sometimes responsible for the physical condition of the child. Epilepsy, idiocy, deaf-mutism are some of the conditions that can be definitely traced to accidents during delivery. It is incumbent upon the obstetrician to make the labor as short and as easy as is consistent with the best interests of the mother.

There are many postnatal factors entering into the making of a vigorous physique. We shall consider these under the different periods of life, as each period has its own peculiar characteristics, and therefore individual needs.

The characteristic of the period of infancy is that there is a lack of development of the inhibitory powers, and a highly irritable condition of the nervous system, the slightest impulses producing very marked results. The infant's surroundings, therefore, should be hygienic to a degree; an abundance of fresh air, quiet, and the proper quantity and quality of food at the proper intervals, are essentials to normal growth and development. Too much can not be said in condemnation of making hot-house plants of infants, It is only because of a kind providence that many of them are not suffocated to death. There is rarely a day in this climate that they may not be carried out, for a while at least. They need plenty of fresh air and are entitled to have it. In the coldest days it must be given them, but under favorable conditions properly warmed. Likewise, the habit of making sudden noise to provoke laughter in the infant is injurious, and to be condemned. It is too exciting to the sensitive nervous systems, and they should be allowed quiet until their inhibitory powers are properly developed. Food in proper quantity and of proper quality, and at proper intervals, is the *sine qua non* of proper growth and development. No one of these conditions is of more importance than the other.

Breast milk is the food *par excellence.* If for any reason this is not to be obtained, cow's milk, properly modified, is the best substitute. The proprietary foods are to be mentioned to be condemned, because they all contain elements that are not found in the natural food of the infant, and which the infant's juices are not prepared to digest. The neglect of these influences is largely responsible for the exceedingly high mortality of the first month and of the first year. Attention to them will tend toward the development of a strong child.

In childhood (from two to six years) the problem is to increase the vital resistance of the child. It is a period of very rapid growth and consequently of lowered resistance. For this reason it is the period when the child is very apt to contract the contagious diseases. Speaking in general terms, a child that has a good resistance offered by vigorous health, will not contract disease even though exposed; but let the resistance be lowered, and the child succumbs readily. This vital resistance may be increased (a) by proper food and hygiene, looking not only after the quantity, quality and intervals of feeding, but also after the dress, exercise, sleep, etc.; (b) by the removal of all sources of infection and all the avenues, such as adenoids, enlarged tonsils, and other pharyngeal affections; and (c) by correcting any existing anemia. As this period is the imitative period of the child, it is just as easy to teach it habits of life that will contribute towards vigorous bodies, as it is for it to learn bad habits of living, and especial care should be exercised toward attaining this end.

The next period of the child's life, from the school age to puberty (six to fourteen), is charaterized by great muscular and nervous development and by comparatively slow growth. The problem then is to bring such influences to bear as will aid and not retard the development of the muscle and nerve. The same attention must be paid to dress, exercise, food, etc., and the additional important element of school hygiene must be taken into consideration. This should include such things as the number of pupils to the room, the amount and character of the work required of the pupils, the supervision of the school by a competent physician, and the special oversight of backward children.

Undoubtedly there are too many children crowded into the rooms of the modern city schools. The necessary inhalation of impure air is injurious to both body and mind. The difficulty can be partially overcome by a careful teacher, if she will so arrange the work as to change the air in the room frequently.

Overburdening of work seems to me to be *the* fault of modern educational methods from both an educational and a physical standpoint, that being here considered. Practically, the same hours of session are required of the child of six as of that of sixteen. Instead of giving the child time to think, and of attempting to train its inductive and deductive faculties, the tendency seems to be to constantly increase the number of studies in the curriculum; and the longer the list of studies that can be displayed in the catalogue the higher the standard of the institution is supposed to be. The ambitious child is compelled to give hours of work at home, preparing for the next day's work in recitation; this demands of the child an expense of nervous energy that it can ill afford, and utilizes time that should be given to outdoor recreation and rest, in order to the proper development of muscle and nerve force. There should be no home studies for a child under ten, preferably under twelve, and the studies during school-hours should be so arranged that the most taxing should come at the time of day when mentality is most active, with those requiring mechanical exertion placed at the period of least activity. Interesting experiments have been made by Griesbach, in Alsace, with the esthesiometer to show the effects of school-work in producing fatigue. The instrument is simply a pair of calipers, whose points can be pressed upon the skin at varying distances apart, the lengh of the intervals being noted on a scale. It was found that as mental fatigue develops the child's ability to distinguish the points as distinct diminishes, so that the distance between them has to be increased. The children were tested at various times during school term, on holidays and during vacation. On holidays the variations in the measurements were inconsiderable, while on school-days, the measurements doubled, and some children showed four times the minimal distance. During the midday recèss recuperation was considerable, but not complete. Even in the morning before school, a large number of the children failed to show as great sensibility as on holidays; probably some of these were night-workers, or were overworked in school the day before. The greatest reduction in sensibility was after mathematics, the least after manual training; thus, close mental work is shown to cause the greatest fatigue, while manual training produces the least.

With little children and with older ones that are making rapid bone growth concentration is possible only for a short time without producing fatigue, and if this fatigue is prolonged it becomes cumulative and complete recuperation is impossible while attending school. Careful attention to the number and character of the different subjects, the length of the lessons and the time of study is necessary.

The daily variation of mental power is such that the curve of mental energy, beginning at a high level in the morning at nine o'clock, rises moderately until ten o'clock, falls at first slowly until eleven o'clock and then very rapidly until the middle of the noon recess; by one-thirty the curve has risen to the eleven o'clock level, but soon falls rapidly, and before three o'clock it is at the twelve o'clock level. In adults there is another rise in the evening between nine-thirty and ten o'clock.

Manifestly the difficult lessons should be taken up early in the morning, and since the real intellectual work is learning the lesson, this study should be done in school under the guidance of the teacher. To learn concentration is more valuable than to learn lessons. The length of the lessons and the study periods should not be more than fifteen minutes in the first two years of school-life, twenty minutes the next two years, twenty-five minutes the fourth and fifth years and forty to forty-five minutes in the high school.

The neglect of these precautions brings about various nervous disturbances in children, ranging from chorea to hysteria, in both the grave and milder forms. In large clinics it has been found that in the months of October and November there are scarcely enough cases of chorea to demonstrate to the classes, while they increase during the school months until the maximum is reached in the spring. Undoubtedly a large part of this is due to overcrowded condition of the schools and the overburdening of the children. There are a great many more cases of insanity among children than the list of inmates at the asylums would indicate. "The overworked brains of school children were complained of as adjuvant causes of lunacy by Peter Frank as early as 1804." We have not improved in this respect during the last hundred years.

Rather an innovation is the school physician. In Bulgaria and in Hungary there are no schools without their physicians. In many of the up-to-date schools of this country they are to be found now. Their duty is to look after the general hygienic surroundings of the children, such as the heating, ventilation, lighting, etc., of the school; to examine the children daily in order to detect any early evidences of disease; and to look into the condition of the nervous systems, the eyes, ears, and pharynges of the children for the detection of the presence of adenoids. Many so-called backward children have been shown to be so only because of the presence of some defect of sight or hearing, that have been corrected by the findings of the school physician; and many epidemics, with their consequent suffering, death and expense, have been avoided through his work.

Children that are really defective should be separated from their more fortunate brethren, and be given special training suited to their individual needs.

Child labor is another factor that bears on the growth and development of the child during this period. Speaking from the standpoint of the child only, he certainly ought to be protected and prohibited from the long hours and close confinement that prevent the proper development of his body and mind, and thereby preclude the possibility of his being his best in the community.

If the child is to build body-muscle and nerve-power during this period, influences must be brought to bear upon him that will overcome those mentioned as detracting from the desired result. Many of the cases of hysteria, melancholia, neurasthenia, epilepsy, idiocy and insanity are directly traceable to the neglect of these influences.

There is but one other period that we must consider, that of puberty and the years immediately following it. It is characterized by the development of the generative organs, the differentiation of the sexes, with slow growth to maturity. The plain duty to the child is to have especial care to those things that will contribute to give them strong bodies, as they are to be the future mothers and fathers of the succeeding generation. As far as the boys are concerned wise counsel is all they need, as they will be more than likely to take care of their exercise, food and sleep, while the danger of overstudy is reduced to an infinitesimal quantity. But they do need wise counsel as to their developing passions and their proper control. They need to have explained the naturalness and necessity of occasional nocturnal emissions, their lack of pathological significance, and the possibility and advisability of virginity. They need to know that many an innocent wife is to-day a hopeless invalid because of a case of gonorrhea in the husband contracted many years before marriage and which he thought was entirely cured long ago. They need to know that one in every twenty Americans has syphilis, and that once contracted may possibly never be eliminated from the system, but shows itself unto the third and fourth generation. They need to know the evil consequences of self-abuse, and that there is a Power that can save them from all of this if they have not will-power enough of their own.

The girls need particular attention during this period, in three particulars especially—dress, education and exercise. It is my conviction after years of mature reflection that a large number of the ills of which so many women of to-day are the victims are the direct results of the neglect of these particulars while they were developing into womanhood. I suppose mothers will continue to constrict the waists of their daughters and crowd their abdominal contents into their pelvic cavities as long as corsets make their dresses hang better and their figures more shapely, in spite of all the protests that are made; but as long as they do they will continue to make invalids of those who might otherwise be vigorous, and keep the gynaecologists of the next generation busy. The dress should be such as will give plenty of freedom to the wearer and cause no undue pressure upon the developing pelvic organs. Displacements of the uterus will thereby be avoided, with all their attendant nervous symptoms, and the girl grow to as vigorous womanhood as her great-grandmother possessed.

The education of the girl at this period of life is not conducive to health. Her curriculum is full and demands of her too much work out of school hours, so that she is deprived of a sufficient amount of exercise and recreation to maintain a stable equilibrium. Her work is done at the expense of her nervous system, and various neuroses result. One particular study demanding unusual care at this time is music. Nothing is more fatiguing to the nervous system than the concentration of every energy necessary to success and required in the long hours of practice. A girl should never be allowed to practice longer than half an hour at a time, and the practice periods should be so arranged as not to interfere with her rest and recreation. She should have some time each day for out-door exercise—horse-back riding, tennis and golf are best. With the proper relation between her exercise, her studies and her dress, every healthy girl at fourteen should grow to vigorous womanhood. If there would be fewer difficult labors, more breast-fed babies, a smaller infant mortality, and a more vigorous succeeding generation.

The physician's relation to the child and the community is to insist upon the latter bringing to bear upon the former all these influences as above indicated that will contribute to a vigorous body, and hence a stronger mentality and morality.

---

WHAT say you to this authentic instance as a proof of the value of physiology teaching in elementary schools? Inspector: "What is our blood composed of and how does alcohol act on it?" Answer: "It is made of one million red insects and one thousand white ones to every drop of blood. Alcohol kills them and sends their carcasses to the front of the body. That is why people who drink alcohol have red noses."

